# Is parity a protective factor in depression and IPV?

**DOI:** 10.1192/j.eurpsy.2023.1352

**Published:** 2023-07-19

**Authors:** B. R. Vargas, V. Valdez, C. Santana

**Affiliations:** Career of Medicine, Universidad Católica de Santiago de Guayaquil, Guayaquil, Ecuador

## Abstract

**Introduction:**

IPV is a public health issue that is often linked to depression. Parity has often been mentioned as a protective factor against depression and suicide attempts. Despite this, parity in Latin America may not be related to positive outcomes for victims suffering from IPV as the stress of taking care of the children can result in a burden and worsen the symptoms of depression for the victim.

**Objectives:**

Determine the impact of Parity in victims suffering from IPV and Depression.

**Methods:**

A descriptive observational study was conducted, at the main Gender Violence Prosecutor’s office: Florida, Guayaquil-Ecuador. UCSG pre-medical students collected the information using Beck test for Depression. The total sample was 239: 195 women, 44 men. It was classified by groups, gender, marital status, children and severity of depression.

**Results:**

The data analyzed showed a higher percentage of Depression from IPV when parity is present.

Severe Depression: Women with children 57 (29%), 8 men with children (18%). Women without children 22 (11%), men without children 6 (14%).

Moderate depression: Women with children 28 (14%), 4 men with children (9%). Women without children 5 (3%), men without children 2 (5%).

Mild Depression. Women with children 25 (13%), 7 men with children (16%). Women without children 6 (3%), men without children 2 (5%).

**Conclusions:**

Although some studies report having children as a protective factor in depression, this did not happen in this study. Financial violence is very common, so the mother does not receive any economic support from the father and has to take care of the children on her own. Social and hormonal factors also play a role, especially in women as they have more children. We believe that mental health clinicians should pay more attention to victims of IPV who have several children, especially in Latin America.

**Disclosure of Interest:**

None Declared

